# The resolution revolution revisited

**DOI:** 10.1107/S2052252526006123

**Published:** 2026-06-15

**Authors:** Edward H. Egelman

**Affiliations:** a Department of Biochemistry and Molecular Genetics University of Virginia Medical School Charlottesville VA22903 USA

**Keywords:** electron cryo-microscopy, cryo-EM, electron cryo-tomography, cryo-ET, resolution

## Abstract

In this issue of *IUCrJ*, Subramaniam, Kühlbrandt & Henderson present an overview of the remarkable progress that has been made in electron cryo-microscopy and electron cryo-tomography.

Though the world does not change with a change of paradigm, the scientist afterward works in a different world.

Thomas S. Kuhn, *The Structure of Scientific Revolutions*

In 2014 Werner Kühlbrandt wrote a Perspective entitled *The Resolution Revolution* (Kühlbrandt, 2014 ▸) about how we were entering a new era in molecular biology where structures of macromolecular complexes could now be determined at near-atomic resolution by electron cryo-microscopy (cryo-EM). The Perspective heralded the publication of a 3.2 Å resolution cryo-EM structure of the mitochondrial ribosome’s large subunit (Amunts *et al.*, 2014 ▸). While this ribosome structure was a spectacular advance at the time, showing that such a complex did not need to be crystallized to reach a resolution that would enable the building of an atomic model, reaching such a resolution is now a daily event using cryo-EM. In fact, there are now ∼1800 ribosome structures at better than 4.0 Å resolution deposited in the Electron Microscopy Data Bank (EMDB), with 36 of these at a resolution better than 2.0 Å. True atomic resolution (which by definition means that individual atoms are resolved) is not reached until 1.2–1.3 Å. If 3.2 Å is near-atomic resolution, than a 1.6 Å ribosome structure (Fromm *et al.*, 2023 ▸) might be called ‘even nearer’ atomic resolution.

Subramaniam, Kühlbrandt and Henderson have revisited the 2014 Perspective in this issue of *IUCrJ* (Subramaniam *et al.*, 2026 ▸), providing a summary of the progress that has been made in cryo-EM and electron cryo-tomography (cryo-ET), as well as highlighting some of the issues and problems that will be likely addressed in the future. While near-atomic resolution structures by cryo-EM were quite rare 15 years ago, they are now the rule, and the exceptions are when such resolutions cannot be reached. The impact on structural biology has thus been profound, and Subramaniam *et al.* discuss this at some length.

What may be less appreciated is that cryo-EM is now beginning to make substantial contributions in fields such as chemistry and materials science. The main tool that has been typically used to understand the structures formed by the self-assembly of peptides and small molecules has been X-ray fiber diffraction, and more recently small-angle X-ray scattering (SAXS) and molecular dynamics (MD) simulations. The structure of tobacco mosaic virus (TMV), the very first virus ever isolated, was solved by X-ray fiber diffraction to 3.6 Å resolution in a monumental effort (Namba & Stubbs, 1986 ▸). Very few people these days use fiber diffraction, since it is far, far easier to simply use cryo-EM. In addition to requiring much less sample and reaching a higher resolution, one can directly generate maps in cryo-EM, while phasing the X-ray intensities collected in fiber diffraction is highly problematic. Thus, a 1.9 Å resolution cryo-EM structure of TMV (Weis *et al.*, 2019 ▸) was generated from only 62 images!

The lanreotide octapeptide self-assembles into tubes that provide a slow-release pharmacological formulation in clinical use, and a fiber diffraction structure was published more than 20 years ago (Valéry *et al.*, 2003 ▸). However, a more recent 2.5 Å cryo-EM structure (Pieri *et al.*, 2022 ▸) of the lanreotide tubes was strikingly different from the original model, and revealed that the asymmetric unit was eight peptides, in eight different environments, with eight conformations. In contrast, the original fiber diffraction model assumed an asymmetric unit of two peptides. Similarly, a naphthalene-modified dipeptide has been shown by cryo-EM (Fig. 1 ▸) to self-assemble into tubes with 18 peptides in the asymmetric unit, in 18 different environments, with 18 different conformations (Bianco *et al.*, 2025 ▸).

Techniques such as SAXS and MD cannot reliably be used to generate atomic models *ab initio*, but these techniques have dominated much of the literature on the self-assembly of peptides and small molecules. It is encouraging that cryo-EM is beginning to supplant those methods (Sonani *et al.*, 2025 ▸). Factors that are currently limiting the applications outside of structural biology include lack of access to high-end instruments and lack of expertise in applying these methods. As cryo-EM matures, both of these obstacles are expected to become less significant.

It is challenging to predict exactly where cryo-EM will be in another ten years after the revolution. As they say in Denmark, ‘Prediction is very difficult, especially if it’s about the future’. Nevertheless, one can be optimistic that advances in hardware and software will continue to be made, enabling a larger and larger community of users to address many fundamental problems in biology and chemistry.

## Figures and Tables

**Figure 1:**
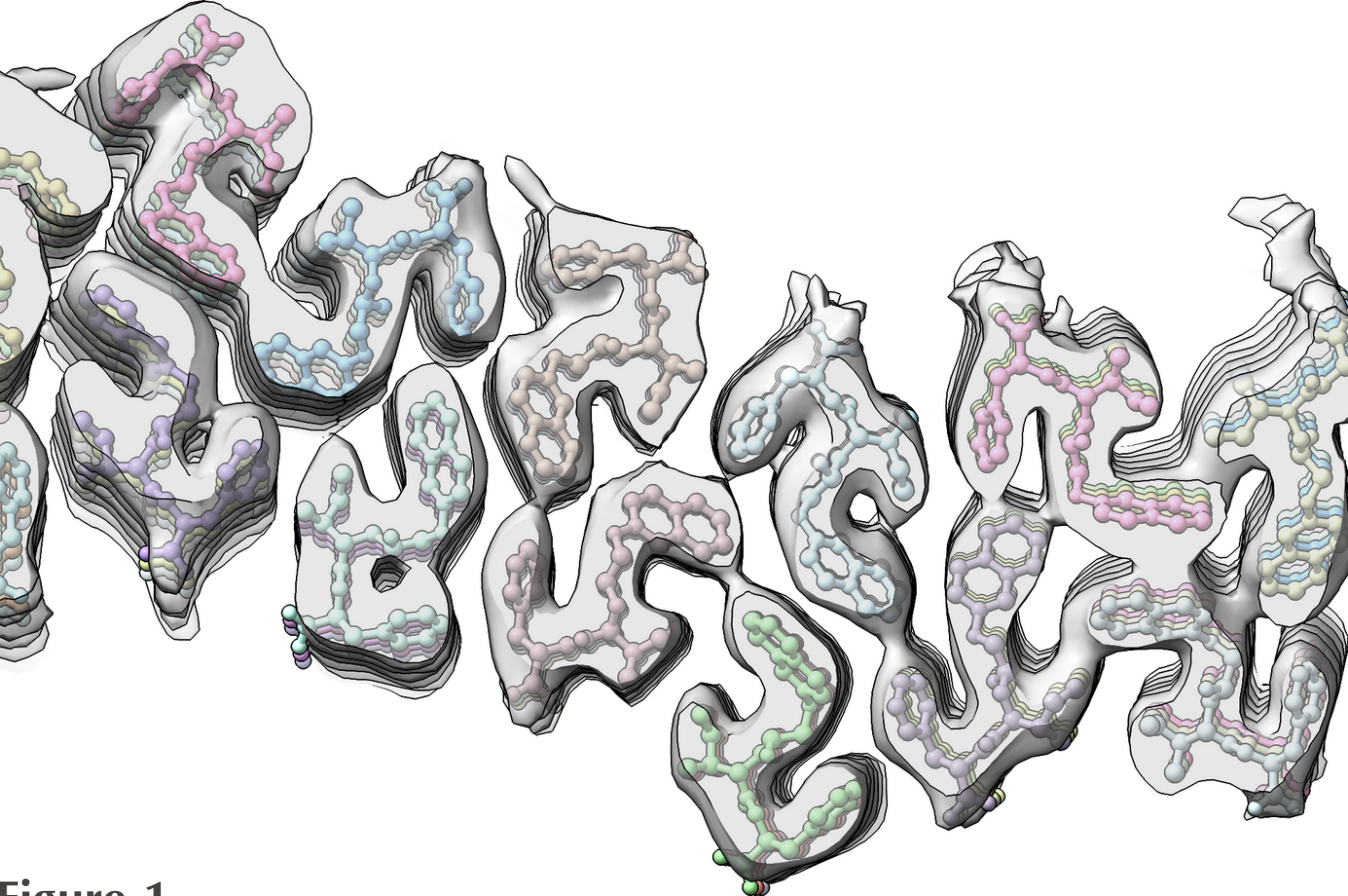
The use of cryo-EM continues to grow outside of structural biology, such as in chemistry and materials science. This figure shows a 3.3 Å resolution cryo-EM map with an atomic model of an assembly of naphthalene-modified dipeptides (isoleucine-phenyl­alanine) (Bianco *et al.*, 2025 ▸). Quite surprisingly, these dipeptides self-assemble into tubes with 18 peptides in the asymmetric unit, with 18 different conformations.
